# Compliance of a Tertiary Centre With Molecular Testing Strategies for Lynch Syndrome in Colorectal Cancer

**DOI:** 10.7759/cureus.73475

**Published:** 2024-11-11

**Authors:** Bennett C Peter, Mohamed Mansour, Kolanu Prasad, Trisha Jha, Sowmya Venkatesan, Madan Jha

**Affiliations:** 1 General Surgery, James Cook University Hospital, Middlesbrough, GBR; 2 Surgery, James Cook University Hospital, Middlesbrough, GBR; 3 Pathology, James Cook University Hospital, Middlesbrough, GBR; 4 Medicine and Surgery, Keele University School of Medicine, Newcastle-under-Lyme, GBR; 5 Colorectal Surgery, James Cook University Hospital, Middlesbrough, GBR

**Keywords:** adenocarcinoma, colorectal cancer, compliance, immunohistocompatibility, lynch syndrome, microsatellite instability (msi), molecular testing strategies

## Abstract

Introduction

Lynch syndrome is one of the most common hereditary cancers associated with germline alterations of DNA mismatch repair genes. Recent advances have shed light on its molecular pathogenesis, leading to the development of various testing strategies. The aim of this study is to evaluate whether all colorectal adenocarcinomas undergo immunohistochemistry (IHC) and microsatellite instability (MSI) testing to identify potential Lynch syndrome patients.

Methods

This study evaluated the compliance of MSI and IHC testing for Lynch syndrome in a tertiary cancer research centre. All patients undergoing colorectal cancer resection between April 2022 and December 2022 were identified from a prospectively maintained register. The histology, MSI, and IHC testing reports for these patients were recorded to check if they were done and reported. Only patients with colorectal adenocarcinoma were included in this study.

Results

A total of 314 patients had colorectal cancer resections. A total of 301 were included in the study, with a median age of 65 years (IQR = 35-95). Thirteen (4.14%) patients did not have MSI and IHC testing reported on the system. Of these, eight (61.53%) did not have an MSI/IHC testing request, four (30.76%) did not have sufficient specimens to be sent for further testing, one (7.69%) had MSI and IHC testing but the final report was yet to be authorized. Out of the eight who did not have the testing done, three had early polyp cancer and one had a goblet cell adenocarcinoma.

Conclusion

According to the data, our hospital has a 97.45% compliance with current guidelines, not considering the insufficient sample for testing and the authorization of the final report. A more comprehensive proforma may be needed to provide a feedback loop for further MSI and IHC testing when entering data into the system. Further auditing is required to check the effectiveness of the proforma in achieving complete compliance.

## Introduction

Lynch syndrome (LS) is the most common hereditary cause of colorectal cancer (CRC), accounting for around 1-5% of all newly diagnosed CRC patients [1-4]. LS is an autosomal dominant condition caused by germline mutation of DNA mismatch repair (MMR) genes, namely, MLH1, MSH2, PMS2, and MSH6, leading to an accumulation of errors during DNA replication [1,5,6]. Patients diagnosed with LS have a 70% lifetime risk of developing CRC and a 15-60% risk of developing extracolonic tumours like endometrial, small bowel, ureter, and renal pelvic tumours [2,5,7,8]. Aggressive and efficient screening methods enable further follow-up, screening, early detection, and treatment of cancers in LS-diagnosed patients and their relatives [5].

Historically, the diagnosis of LS was based on comprehensive history taking and utilization of the Amsterdam and Bethesda criteria. These, however, had a very low sensitivity, leading to underdiagnosis and missed opportunities for surveillance [3]. Currently, CRC is further analysed using three main molecular pathways: chromosomal instability (CIN), microsatellite instability (MSI), and CpG island methylator phenotype (CIMP) pathway [9]. LS tumours tend to follow the MSI pathway and loss of expression of MMR proteins, which can be identified on immunohistochemistry (IHC) testing [3,10]. The loss of this corrective system enables dysregulation of protein function and cellular metabolism, leading to mutations and eventually carcinogenesis [11]. This has led to the development of alternate screening guidelines, initially put forward by the Evaluation of Genomic Applications in Practice and Prevention (EGAPP) in 2009, followed by the National Comprehensive Cancer Network (NCCN) in 2014 and the National Institute for Health and Care Excellence in 2017, where all newly diagnosed CRC were to undergo LS screening [5].

The purpose of this paper is twofold: first, to evaluate the compliance of a regional tertiary centre with the national guidelines; second, to identify potential challenges in the screening protocol and ways to improve them.

## Materials and methods

In the north-eastern region of the UK, MSI and IHC testing is carried out at the Cellular and Molecular Pathology Laboratory in Newcastle. All CRC-diagnosed specimens from the NHS trust in the region are transported to this pathology centre, and the request and clinical history of the patient are attached. Before December 2021, tumour testing was only IHC-based. Currently, it is being tested on a molecular basis, thereby simultaneously testing for MSI, KRAS and BRAF genes.

In our trust, CRC staging is done based on the American Joint Committee on Cancer (AJCC) tumour, node, and metastasis system, 8th edition. Following diagnosis of colorectal adenocarcinoma, all samples are then packaged, sealed, and sent for MSI and IHC testing. The results are then cross-checked and sent back to our institution, following which they are uploaded onto the investigations software, which is accessible to the treating surgeon. Following the detection of MSI instability and detection of loss of expression of MMR protein by IHC testing, genetic counselling is advised for the patients due to the possibility of detection of LS.

We conducted a retrospective study on all patients who were newly diagnosed with CRC between April 2022 and December 2022 in a major trauma centre. Identification of patients for the study was done from a prospectively maintained register from the Department of Histopathology. Patients of all age groups with newly diagnosed colorectal adenocarcinoma were included in this study. Both emergency and elective resections were also incorporated into this study. Patients with recurrence or without biopsy-proven adenocarcinoma were excluded. A review of the histopathology results and further MSI/IHC testing were conducted from an electronic database. Data collected included patient demographics (age and gender) and clinicopathological features (location of tumour, surgery performed, differentiation of tumour, and adjuvant or neo-adjuvant therapies). Data collected were stored in hospital-based computer systems with intranet connection.

The primary outcome was to ascertain if MSI and IHC testing were done for all newly diagnosed colorectal adenocarcinomas. The secondary outcome was to look at potential challenges in reaching the desired compliance and find solutions for the same.

## Results

A total of 314 patients with newly diagnosed CRC were identified. After implementing the inclusion and exclusion criteria, 301 patients were included in this study. Patient demographics are summarized in Table 1.

**Table 1:** Patient demographics and sites of colorectal tumours referred for MSI and IHC testing. MSI: microsatellite instability; IHC: immunohistochemistry.

Features	n (%)
Gender	
Male	170 (56.48)
Female	131 (43.52)
Age	
<50	12 (24.49)
50-60	49 (20.42)
>60	240 (79.73)
Tumour site	
Rectum	109 (36.21)
Sigmoid	59 (19.60)
Caecum	35 (11.63)
Ascending colon	36 (11.96)
Transverse colon	24 (7.97)
Descending colon	12 (3.99)
Splenic flexure	10 (3.32)
Rectosigmoid	7 (2.33)
Hepatic flexure	6 (1.99)
Synchronous tumours	2 (0.66)
Anal canal	1 (0.33)
Status	
Alive	254 (84.39)
Deceased	52 (17.28)

The most common site of CRC was the rectum (36.21%), with the second most common being the sigmoid (19.60%). Two patients had synchronous tumours at the ascending colon and splenic flexure and underwent subtotal colectomy. Of the 301 patients included, 254 (84.39%) are alive and 52 (17.28%) are deceased.

Of the 301 patients who were diagnosed with CRC, 13 (4.31%) did not have MSI/IHC testing for LS. All the other 288 patients (95.68%) had both MSI and IHC testing done and reported. Of the 13 who did not have molecular testing, eight (61.53%) did not have an MSI/IHC testing request sent on the system, four (30.76%) did not have sufficient specimens for further testing as these patients were put on a best supportive care or palliative care treatment and the initial biopsy specimen had been used up for diagnosis, and one (7.69%) sample had been sent but the report was not finalized and hence was not on the system. Of the eight samples that were not sent for testing, three samples showed early polyp cancer and one was a goblet cell adenocarcinoma. The sample which was sent but not reported on the system was not included in the compliance calculation as the protocol was followed but the response was not received from the laboratory. Additionally, those samples not sufficient for further testing were excluded from the compliance calculation. Removing these patients, our institution has a 97.45% compliance with the National Institute for Health and Care Excellence (NICE) guidelines in terms of molecular testing strategies for LS in CRC patients. The chance of early polyp cancers being missed to be sent for MSI/IHC testing is an indication to put prompts on the reporting system so as not to be missed.

## Discussion

Our NHS trust provides colorectal services to a catchment area of around 1.5 million patients. Of all the newly diagnosed CRC in our institution, 97.45% underwent further MSI/IHC testing for the detection of LS, closely adhering to the national guidelines. Though this seems an optimistic result, every effort needs to be made to have complete compliance with the guidelines. This enables early detection and active surveillance of the population [3,12,13]. Two of the 301 patients sent for testing were diagnosed with LS. One of them was known to have LS and was on surveillance due to the diagnosis of a near relative with LS. This patient developed CRC at the age of 61 and had a good outcome due to early detection thereby emphasizing the necessity of testing for LS.

Detection of LS has always been a major topic of discussion due to the interventions and surveillance protocol for LS. Earlier methods of diagnosis involved comprehensive family history and scoring systems like the Bethesda guidelines and Amsterdam criteria to predict the chances of LS. However, these scoring systems have proved to have low sensitivity, leading to frequent underdiagnosis and missed opportunities [14,15]. This mainly occurs due to poor documentation, failure to recall three generations of family history by the patient, and atypical presentation of CRC at a later age. This is evident in a study done by Church et al., which involved the implementation of an updated questionnaire, organizing awareness courses and set-up of prompts following poor history taking and documentation for scoring using the Amsterdam II criteria. Four years after the implementation of the change, it was shown that the accuracy of CRC history taking for scoring remained the same. One major observation was the lack of recall by the patients with respect to family history [15]. In another study conducted by Grover et al., the effectiveness of history taking of physicians in an oncology clinic identified only about 67% congruence in accurately recording family history as per the Amsterdam criteria [16]. This leads to a missed opportunity to diagnose LS, making the criteria less reliable for detection of LS.

Lynch et al. described an observational cohort study reporting atypical presentations that can deter the diagnosis of LS. One of the observations was the late age of cancer onset, which, according to the Amsterdam criteria, would rule out further testing for LS [6]. This can be detrimental to the patient and their family as further surveillance would be cancelled. However, molecular testing of the CRC would help diagnose LS and undergo further management and follow-up.

A study similar to ours was done in the US by Hill et al., in 2014, which showed 75% compliance with the universal guidelines since the adoption of these guidelines in 2009 [5]. According to the study, the main contributing factors towards non-compliance were a lack of specimens for testing and a lack of awareness to send samples for further investigation. Hill et al. mentioned that effective feedback communication was necessary to ensure proper referral of specimens when diagnosed with CRC to improve compliance. This would involve completion of a form to send samples for molecular testing when uploading a CRC histopathology result onto the system.

According to Overbeek et al., one recommendation to improve compliance for molecular testing strategies is to introduce electronic reminders for pathologists when reporting CRC on the system. They have shown a 2.8 times improved rate of detecting LS when implementing electronic reminders for the pathologist [14]. It also showed a 23% increase in requests for molecular testing on diagnosis of CRC. As most hospitals provide electronic reports, it makes it easier to input an electronic prompt to send for molecular testing on uploading a result of CRC. This comes with an increase in the cost of detection of LS by implementing additional software/electronic systems [14].

Another study from the US done by Singh et al. showed an overall increase in the number of CRC cases being referred for LS diagnosis by the implementation of an automated system CLEAR LS (Closed Loop Enhanced Assessment and Referral for Lynch Syndrome). This system's approach incorporates pathology screening, cancer genetics referral, and computer programming to automatically send all CRC-reported cases for IHC and MSI testing. They additionally showed a reduction in the overall cost of diagnosing and treating patients with LS due to early diagnosis and intervention [17].

This study is limited to a single centre and data need to be shared between centres to maximize and compare compliance with the diagnosis of LS. Additionally, this study is a retrospective design and is prone to selection bias. A more prospective analysis of samples being sent for MSI and IHC testing is required. The study period is limited to nine months and needs a longer-term assessment of trends and seasonal variations in MSI and IHC testing. However, this study has recruited a large cohort of patients due to our centre being a tertiary cancer research centre.

## Conclusions

Molecular testing strategies adopted in recent years have proved to be essential in the early detection of LS in CRC patients. This is associated with early surveillance, detection, and management of hereditary tumours associated with LS. Our institution has reported 97.45% compliance with the guidelines, but all efforts are to be made to ensure complete compliance with guidelines. One method is to implement electronic reminders for reporting pathologists to send samples for further testing, thereby ensuring a robust system to ensure complete compliance. Further audit is required in this regard to detect the compliance of our institution with the national guidelines.
